# The frequency of Epstein-Barr virus among hemodialysis patients, Ahvaz, Iran

**Published:** 2019-02

**Authors:** Rahil Nahid Samiei, Shahab Mahmoudvand, Somayeh Shokri, Manoochehr Makvandi, Heshmatollah Shahbazian, Roya Pirmoradi, Shokouh Shayanpur, Kimia Makvandi, Sepideh Nowrozi

**Affiliations:** 1 Department of Virology, School of Medicine, Ahvaz Jundishapur University of Medical Sciences, Ahvaz, Iran; 2 Infectious and Tropical Diseases Research Center, Ahvaz Jundishapur University of Medical Sciences, Ahvaz, Iran; 3 Department of Nephrology, Golestan Hospital, Ahvaz Jundishapur University of Medical Sciences, Ahvaz, Iran; 4 Nephrology Department, Imam Khomeini Hospital, Ahvaz Jundishapur University of Medical Sciences, Ahvaz, Iran; 5 School of Medicine, Ahvaz Jundishapur University of Medical Sciences, Ahvaz, Iran

**Keywords:** Epstein-Barr virus, Hemodialysis patients, Enzyme-linked immunosorbent assay

## Abstract

**Background and Objectives:**

Epstein-Barr virus (EBV) has infected more than 90% of adults worldwide. EBV infection is asymptomatic in healthy individuals and is controlled by a robust immune response while in individuals with weakened immunesystems including Hemodialysis (HD) patients and transplant recipients leads to serious illnesses. This study was aimed to investigate the frequency of EBV among the HD patients.

**Materials and Methods:**

The cross-sectional study was carried out on 84 HD patients. These sera were checked for anti-EBV (VCA) IgG Ab assessment using enzyme-linked immunosorbent assay (ELISA). The DNA was extracted from the sera samples and tested for EBV DNA using nested PCR.

**Results:**

52/84 (61.9%) of HD were males and 32/84 (38.1%) were females. The average age of participants was varying from 18 to 85 years while the mean age was 52 ± 1.57 SD years. 81 of 84 (96.42%); including 49/52 (94.23%) male and 32/32 (100%) female, were positive for anti-EBV (VCA) IgG antibody while 3 (3.58%) were negative. No significant differences were observed between the subjects regarding gender (P=0.28). EBV DNA was detected in 7 (8.33%) individuals, including 6 (11.53%) and 1 (3.12%) in male and female, respectively (P=0.24).

**Conclusion:**

Our study results showed that high prevalence of anti-EBV (VCA) IgG antibody (96.42%) were observed among the HD patients. Although the status of EBV latency was not performed, but it seems many of these patients are at risk of EBV-reactivation during the organ transplantation. As a result, it is recommended that the detection of EBNA-1 gene as a marker of EBV latency should be implemented for all HD patients to prevent EBV reactivation during organ transplantation.

## INTRODUCTION

Epstein-Barr virus (EBV), a member of the herpesvirus family, is one of the most common human infections that infects more than 95% of the world populations (1). It has been implicated in several diseases, including infectious mononucleosis, African Burkitt lymphomas (BL), Hodgkin's lymphoma, B-cell lymphomas of immunocompromised, nasopharyngeal carcinomas (NPC), stomach cancer. Most individuals contract EBV infection in early adulthood (2). The transmission of EBV occurs mainly via contact with saliva. However, the virus can be transmitted through sexual contact, blood transfusions, and organ transplantations (3, 4). Hemodialysis (HD) is a process of purifying the blood of a person whose kidneys are not working normally, especially patients in end-stage renal failure. Renal transplantation is considered the treatment of choice for these patients (5). Patients in end-stage renal failure have severe alterations in cell mediated immunity that increase their risk of contracting opportunistic viral infections such as EBV infection (6). EBV is most likely to cause problems when the immune system is suppressed by disease (7). Therefore, immunocompromised hemodialysis patients and renal transplant patients are at high risk for EBV infection. Like all herpesviruses, after primary infection, EBV establishes a persistent infection in almost all infected host, which may be responsible for the development of several diseases. The virus can cause a latent infection within B lymphocytes, allowing the virus to evade the host immune response (8). Latent EBV in B cells can be reactivated to switch to lytic replication, especially in individuals with weakened immune systems (9, 10). Post-transplant lymphoproliferative (PTLD) is one of the most important EBV-associated complication. PTLD is a severe complication of solid organ that occur after a transplant, a leading life-threatening malignancy in the transplant population. It can develop in people who are taking immunosuppressive drugs to prevent rejection of an organ. EBV is the main driver of PTLD, particularly in patients with impaired immunosurveillance against EBV, and is contributes to the pathogenesis of PTLD in more than 70% of cases (11, 12). Therefore, the PTLD occurrence is preceded by increased number of latently infected B-cells and EBV reactivation (8). The virus-associated various tumors mainly encompass latently infected B-lymphocytes in which EBV-encoded growth-transformation-associated proteins are expressed (13). Therefore, to prevention of EBV complications, hemodialysis patients should be routinely tested for EBV Viral Capsid Antigen (VCA) IgG antibody and EBV DNA before transplantation. The aim of this study was to evaluate serological and molecular status of EBV infection in the HD patients.

## MATERIALS AND METHODS

### Samples collection.

In this cross-sectional study, the sera of 84 (including 32 females and 52 males) HD patients who referred to Golestan hospital were collected during October 2014 to November 2014. Their sera were stored at −20 before use.

### EBVspecific IgG antibody detection by Enzyme-linked Immunosorbent Assay (ELISA).

Sera were tested for the presence of EBV Viral Capsid Antigen (VCA) IgG antibody by ELISA kit (DIAPRO, diagnostic, Milan, Italy) according to the manufacturer's instruction. Cut-off was defined with positive and negative control sera that were included in each assay, according to the manufacturer's instruction.

### DNA extraction.

DNA was extracted from serum using High Pure PCR Template Preparation kit (Roche, Germany) according to the manufacturer's instructions. The extracted DNA was reanalyzed for the presence of the EBVDNA using nested PCR.

### EBV DNA detection by nested PCR.

All samples were subjected to nested PCR for detection of EBVDNA, using specific primers, EBV outer Forward: 5′-TGGAAAC CCGTCA CTCTC-3′, Reverse: 5′-TA-ATGGCATAGGTGGAATG-3′ primers were used for first run and EBV Inner Forward: 5′-AGGGAT-GCCTGGACACAAGA-3′, Reverse: 5′-GCCTCG-GTTGTGACAGAG-3′ primers for second run (14). Nested PCR was performed in a total volume of 25 μL, containing the 5 μl extracted DNA, 0.2 μl MgCl_2_ 25 mM,0.5 μl deoxyribonucleotide triphosphates solution 10 μM (Roche, Germany), 2.5 μl PCR buffer 10× (Roche, Germany), 1U Taq DNA polymerase (Roche, Germany), 1 μl from each primer, and 14.5 μl distilled water. Following thermal conditions was carried out: For the first round of PCR program: initial incubation at 94°C for 5 min followed by 35 cycles including 95°C for 45 sec, annealing at 50°C for 45 sec and extension at 72°C for 45 sec, and final extension at 72°C for 10 min. The second round PCR was the same as the first round PCR but annealing temperature was 53°C.

Then PCR product was subjected to 2% agarose gel stained with SYBR Safe DNA gel stain. The bands were visualized using UV transilluminator.

### Statistical analysis.

The collected data were analyzed with SPSS 16 package program (SPSS Inc., Chicago, IL, USA) and Fisher's exact test was used to assess the rate of EBV antibody and EBVDNA among the gender detection calculate. A P-value below 0.05 was considered statistically significant.

## RESULTS

Of 84 HD patients 52 (61.9%) were male and 32 (38.1%) were female. The average age of participants was varying from 18 to 85 years with mean ages of 52 ± 1.57 SD years. 81 (96.42%) subjects were shown positive for anti-EBV IgG antibody while 3 (3.58%) were negative. Overall the prevalence of anti-EBV IgG was 96.42%. Among them 49/52 (94.23%) male and 32/32 (100%) female were positive for anti-EBV IgG antibody. No significant differences were observed between the subjects regarding gender (P=0.28). EBV DNA was detected in 7/84 (8.33%) (Table 1).

**Table 1.** EBV DNA distribution in male and female HD patients

**Gender**	**Positive**	**Negative**	**P-value**
Male (n=52)	6 (11.53%)	46 (88.47%)	
Female (n=32)	1 (3.12%)	31 (96.88%)	P=0.24
Total (n=84)	7 (8.33%)	77 (91.67%)	

## DISCUSSION

Immunosuppressed patients may flunk to create an effective immune response against EBV. This may result in to a persistent infection, which may be account for the creation of PTLD, Burkitt lymphoma, and Large B cell lymphoma, are well-known life-threatening complication of solid organ transplantation (15, 16).

In the current study, the rate of positivity for anti-EBV (VCA) IgG antibody was 81/84 (96.42%). Also, EBV DNA was detected in 7/84 (8.33%). The rate of EBV DNA among the male (11.53%) and female (3.12%) was not significant (P=0.24). No significant differences was observed in EBV IgG antibody in male (94.23%) and female (100%) individuals (P=0.28).

In study conducted by Verghese et al in USA, EBV DNA was detected in 32/95 (34%) pre-transplant (17). Study performed by Nikoobakht et al. demonstrated that the frequency of positive EBV DNA in pre-transplant saliva samples was 44.1% increasing to 67.6%, after transplantation (18). This controversy in EBV DNA positive cases may come from the differences in genetic background of the patients and differences in EBV distribution in different country. The prevalence of anti-EBV IgG in hemodialysis patients in Croatia was 98% (19). In another study performed in Cyprus, the prevalence of EBV IgG antibodies among hemodialysis patients was 94% (20). Saghafi et al. reported that the prevalence of EBV IgG antibody is 100% among adult potential donors and recipients (21). Our results were consistent with recent studies. In study conducted by Beladi Mousavi, EBV IgG antibody was positive in 70% of recipients and 52% of donors but there was no statistically significant difference between males and females (p=0.94) (22). The present study found no significant difference in EBV IgG antibody seroprevalence between genders, which is similar to the result of study conducted by Beladi Mousavi among hemodialysis patients. In study performed in Iran, 15.5% of the renal transplant recipients were positive for EBV infection (23).

It is noteworthy that EBV has been identified as a cofactor in the pathogenesis of a significant proportion of PTLD (11). Furthermore, the virus may lead to graft rejection (24, 25). It has also been reported some of EBV associated lymphomas after solid organ transplant (26). Currently, there is no licensed vaccine to prevent EBV infection. Several clinical trials for a vaccine were conducted in the world (27). Therefore, it seems that screening is required to reduce the further complications of EBV infection in hemodialysis patients. Detection of EBNA-1 protein or EBNA-1 gene is marker of the EBV latent infection. There are serologic tests for assessing the status of EBV prior infection and reactivation. Antibodies to EBNA-1, Epstein–Barr virus nuclear antigen 1, can be measured to help diagnose prior infection while early antigen-diffuse (EA-D) IgG, can act as a marker of EBV reactivation (28). As well as, EBV viral load test, can help as a predictor of EBV-related post-transplant lymphoproliferative disorders in transplant patients (29). Treatment with immunosuppressive drugs may lead to EBV reactivation. Thus, to prevent reactivation before starting immunosuppression therapeutic strategies such as valganciclovir should be used in immunocompromized patient populations who are at high risk of EBV reactivation (30).

In sum, the results of the present study confirm a high prevalence of EBV IgG seropositivity among HD patients. As a result, due to the clinical importance of EBV reactivation and its serious complications in immunosuppressed patients, it is suggested that further studies with more sample size to be conducted to determine the role of EBV in hemodialysis patients. Although the status of EBV latency was not evaluated in the present study but it requires further investigation. Thus, it is recommended that the detection of EBNA-1 antibody, EBV viral load and EA-D IgG as a marker of EBV latency and EBV reactivation should be implemented for all HD patients prior to kidney transplant to prevent EBV reactivation and EBV complication consequences.
